# Efficacy and safety of decitabine in combination with G-CSF, low-dose cytarabine and aclarubicin in newly diagnosed elderly patients with acute myeloid leukemia

**DOI:** 10.18632/oncotarget.3361

**Published:** 2015-01-31

**Authors:** Jianyong Li, Yaoyu Chen, Yu Zhu, Jianfeng Zhou, Yanli Xu, Yan Li, Kang Yu, Ling Pan, Jianmin Wang, Jiahua Ding, Jian Gu, Shanhua Zhou, Jinning Shi, Ming Hong, Ji Xu, Liangqin Pan, Limin Duan, Run Zhang, Sujiang Zhang, Huayuan Zhu, Hua Lu, Peng Liu, Hongxia Qiu, Hanxin Wu, Sixuan Qian

**Affiliations:** ^1^ First Affiliated Hospital of Nanjing Medical University, Jiangsu Province Hospital, Nanjing, China; ^2^ Collaborative Innovation Center for Cancer Personalized Medicine, Nanjing Medical University, Nanjing, China; ^3^ Tongji Hospital affiliated to Tongji medical college of Huazhong University of Science and Technology, Wuhan, China; ^4^ Nanjing Hospital Affiliated to Nanjing Medical University, Nanjing, China; ^5^ First Affiliated Hospital of China Medical University, Shenyang, China; ^6^ First Affiliated Hospital of Wenzhou Medical College, Wenzhou, China; ^7^ West China Hospital of Sichuan University, Chengdu, China; ^8^ Changhai Hospital, Secondary Military Medical University, Shanghai, China; ^9^ Zhongda Hospital, Southeast University, Nanjing, China; ^10^ Clinic Medical College of Yangzhou University, Yangzhou, China; ^11^ Zhongshan Hospital, Fudan University, Shanghai, China; ^12^ Jiangning Hospital, Nanjing, China

**Keywords:** D-CAG, elderly patients, AML

## Abstract

**Purpose:**

This prospective phase II, open label, study was designed to assess the efficacy and safety of D-CAG induction treatment for elderly patients with newly diagnosed AML.

**Experimental Design:**

All patients in this study were treated with decitabine of 15 mg/m^2^ for 5 days and G-CSF for priming, in combination with cytarabine of 10-mg/m^2^ q12h for 7 days and aclarubicin of 10 mg/day for 4 days (D-CAG).

**Results:**

Among 85 evaluable patients, overall response rate (ORR) and complete remission (CR) were 82.4% and 64.7%, respectively, after 1 cycle of therapy. The ORR in patients aged <70 years was 83.0% and 81.6% in patients aged ≥70 years. There was a significantly longer median overall survival (OS) in patients with response (16 months) than in those without response (7 months, *p<* 0.0001). The OS for patients aged ≥70 years and 60-69 years was 10 months and 12 months, respectively (*p*=0.4994). The two-year OS probability was 19.2% and the twenty-month survival rate was 33.8%. Induction mortality of D-CAG treated elderly patients with AML is 4.4%.

**Conclusion:**

D-CAG regimen was well tolerated and showed a promising clinic efficacy in elderly patients with AML (≥70 years).

## INTRODUCTION

Acute myeloid leukemia (AML) in the elderly population (aged ≥70 years) has a high incidence with >20 cases per 100000 patients every year [1]. The combination of cytarabine and anthracycline remains the cornerstone of chemotherapy in those patients and this treatment confers remission in up to 60% of elderly patients with *de novo* disease and 40% with secondary AML [2]. However, many elderly patients develop disease recurrence and die of either disease progression or associated complications. Therefore, those strategies including intensive chemotherapy, low-dose cytarabine, and palliative care are not the appealing approaches for most elderly patients with AML [3].

The combination of novel drugs and low-intensity chemotherapy were developed to reduce the early mortality and improve the benefit-risk ratio for long-term survival in elderly AML patients. Among them, DNA hypomethylating agents showed better single-agent clinical activities in both myelodysplastic syndromes (MDS) and AML, with high survival rates and low risks associated with induction chemotherapy [4-6]. Decitabine, a hypomethylating agent, inhibits DNA methyltransferase 1(DNMT1) [7], reactivates of silenced genes, and induces differentiation of leukemia cells [8]. In clinic, decitabine treatment decreased methylation and induced a better clinical response [9]. Several clinical trials indicated that low-dose of decitabine (15-20 mg/m^2^/day, the optimal demethylation dose *in vitro*) led to a significantly better response rate [4-6, 10-12]. Although the mechanistic basis for the clinical activity of decitabine has not been precisely defined, the clinical and biologic activities of decitabine are very encouraging [10, 12, 13].

Our previous studies revealed that the standard-dose of CAG regimen consisting of low-dose cytarabine and aclarubicin in combination with granulocyte colony-stimulating factor (G-CSF) priming as an induction therapy was well-tolerated by patients and led to a complete remission (CR) rate of 50.0% in patients aged ≥ 70 years, and a CR rate of 40.0% in patients with unfavorable cytogenetic aberrations [14]. In this study, we conducted a phase II trial that evaluated the efficacy and safety of decitabine in combination with modified CAG regimen (low-dose cytarabine and aclarubicin) in elderly patients with AML.

## RESULTS

### Patient characteristics

**Figure 1:**
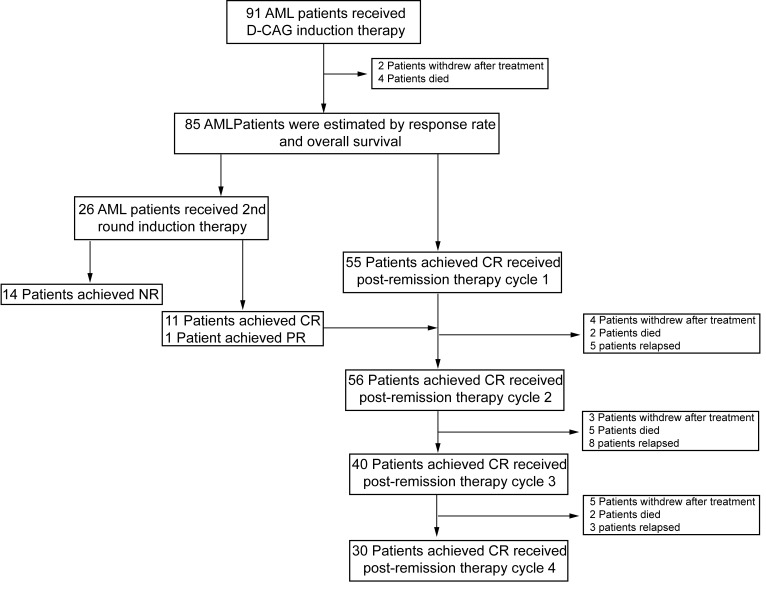
Enrollment and outcomes 91 AML patients were treated with decitabine for 5 consecutive days (day 1-5) and G-CSF (day 0-9) for priming combined with cytarabine (day 3-9), aclarubicin for 4 days (day 3-6) (D-CAG). Up to two cycles of induction therapy were allowed if response was not achieved. Patients who achieved CR accepted the next cycle treatment for recovery of hematopoiesis and resolution of all toxicities. Patients who did not achieve CR or PR after the second cycle of induction therapy were offered alternative therapies. Post-remission therapy consisted of 4-6 cycles. Treatment was continued until relapse or progressive disease, death, or unacceptable toxicity occurred, or patients/physicians requested the discontinuity.

From October 2010 through March 2013, a total of 91 patients with age from 60 to 87 years (median age: 68) were enrolled onto the study (Fig. 1). Among those cases, 42 patients (46.2%) were ≥ 70 years of age. Baseline characteristics are shown in Table 1. According to WHO classification, 91 patients had AML (53 *de novo*, 38 secondary AML). Karyotype included 55 patients (60.4%) with diploid cytogenetics, 15 (16.5%) with chromosome −5/−7 or complex cytogenetic abnormalities, and 12 (13.2%) with other miscellaneous chromosomal abnormalities. Overall, 70 patients (76.9%) were considered ineligible for intensive chemotherapy (aged >70 years, ECOG PS ≥3, comorbidity score ≥2, or adverse cytogenetic).

**Table 1:** Patient Characteristics

Characteristics	No.		%
Age,years			
median		68	
range		60-87	
Male sex	56		61.5
Diagnosis (WHO)			
M1	11		12.1
M2	45		49.5
M4	7		7.7
M5	16		17.6
M6	12		13.2
AML, *de novo*	53		
sAML	38		
Secondary MDS	26		
Secondary myeloproliferative disorders	3		
Secondary immune disease	2		
Secondary other tumor	5		
Complicated solid tumor	2		
WBC, ×10^9^/L			
Median		4.1	
Range		0.16-239.9	
Platelets, ×10^9^/L			
Median		49	
Range		6-974	
Hb, ×10^9^/L			
Median		73	
Range		34-132	
BM blasts			
Median		47	
Range		22-95.2	
karyotype-risk (n=)*			
Good Intermediate	1 66		1.1 72.5
Poor	15		16.5
Unavailable	9		9.9
FLT3 mutational status			
Wild-type FLT3	48		52.7
FLT3-ITD mutation	6		6.6
Missing	37		40.7
NPM1 mutational status			
Wild-type NPM1	39		42.9
NPM1 mutation	15		16.5
Missing	37		40.7
CEBPα mutational status			
Wild-type CEBPα	48		52.7
CEBPα mutation	6		6.6
Missing	37		40.7

Abbreviations: BM: bone marrow; WBC, white blood count; ITD,internal tandem duplication; *Cytogenetic groups were defined as follows: favourable –t(8;21), inv(16), irrespective of the presence of other abnormalities; adverse – monosomy 5, monosomy 7, del(5q), abnormal 3q, complex (5 or more chromosomal abnormalities); intermediate – all other abnormal karyotypes, normal karyotype.

### Treatment outcomes

### Response rates for all patients or selected subgroups

**Figure 2:**
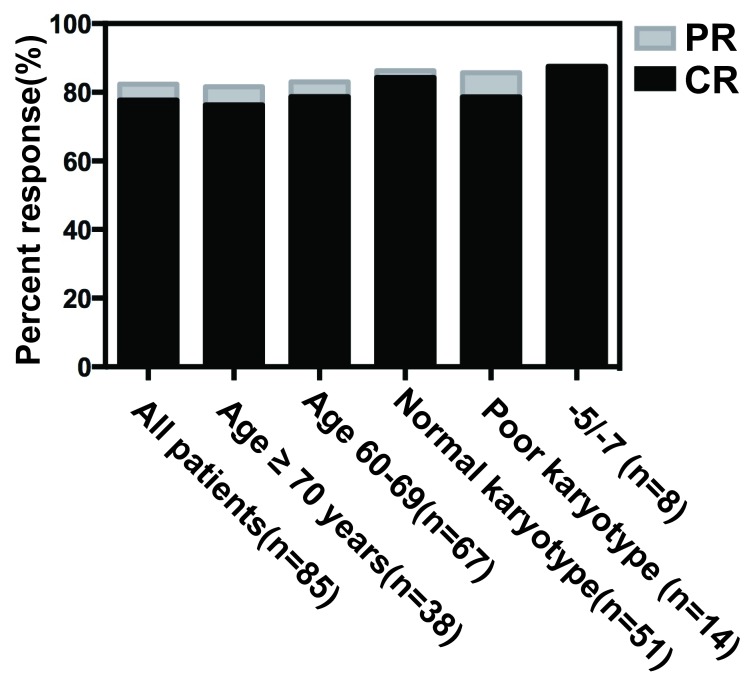
Response rates for all patients or selected subgroups Percent response was noted by each bar graph. CR was noted in black, and additional patients with partial remission were noted in gray. The response rates for all patients, patients below or above the age of 70 years, patients with AML, and selected cytogenetic subgroups were shown respectively.

Patient responses were summarized in Fig. 2. There were 85 patients evaluable for response assessment following the D-CAG induction therapy and had an overall response rate (ORR) of 82.4% after the first cycle. Among them, 55 (64.7%) achieved CR and 15 (17.7%) had PR. After the second cycle, 11 patients with PR acquired CR. The ORR in patients with aged <70 years was 83.0% and 81.6% in patients with aged ≥70 years. The CR in patients with presenting WBC>30 × 10^9^/L (range, 30.9-239 × 10^9^/L) was 68.4% (13 of 19). The CR was observed in all cytogenetic subsets. For patients with a normal karyotype, 84.3% (43 of 51) achieved CR. 18 of 63 patients with CR (28.6%) had abnormal karyotypes at baseline with complete cytogenetic responses (CCyR, 0% abnormal metaphase) of 77.8% (14 of 18). Patients with complex karyotypes (defined as ≥3 abnormalities) had an ORR of 92.9% (CR, 78.6%) and 7 of 8 patients with chromosome −5/−7 (87.5%) achieved CR.

### Overall survival of elderly patients with AML

**Figure 3:**
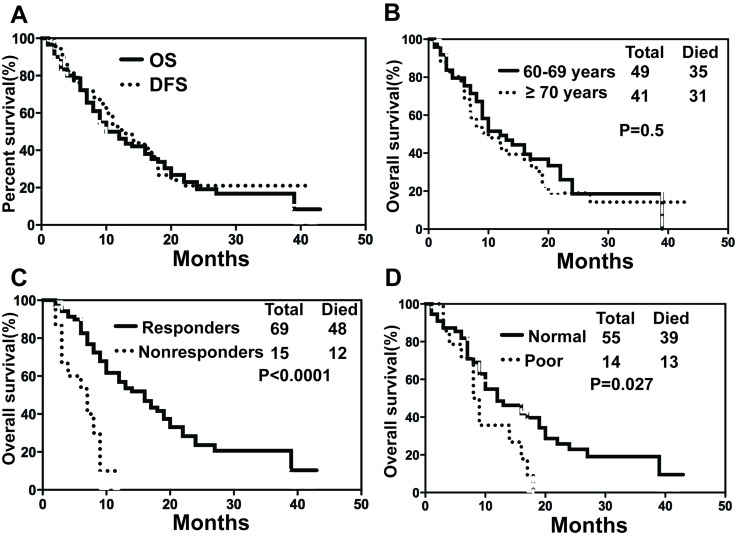
Overall survival (A) all patients with AML, (B) patients ≥ 70 years old or within 60-69 years old, (C) responder or non-responder, (D) normal karyotype or poor karyotype

Upon final analysis (April 30, 2014), the median OS for all patients was 10 months (range, 1-42 months) with a 1- and 2-year OS probability of 46.23% and 19.16%, respectively (Fig. 3A). Median OS was 10 months (range, 1-42 months) for patients aged ≥70 years and 12 months (range, 1-39 months) for patients aged 60-69 years (*p*=0.4994, 95% CI=0.7194 to 1.966) (Fig. 3B). There was a significantly longer median OS in responders than in non-responders (16 *vs* 7 months, *p*<0.0001, 95% CI= 0.0397 to 0.3198) (Fig. 3C). However, survival was shorter for patients with poor karyotype (−5/−7 or complex karyotype) in comparison with patients with normal karyotype (*p*= 0.0269, 95% CI= 0.1788 to 0.9007, Fig. 3D).

### Gene mutation affected the overall survival of elderly patients with AML

**Figure 4:**
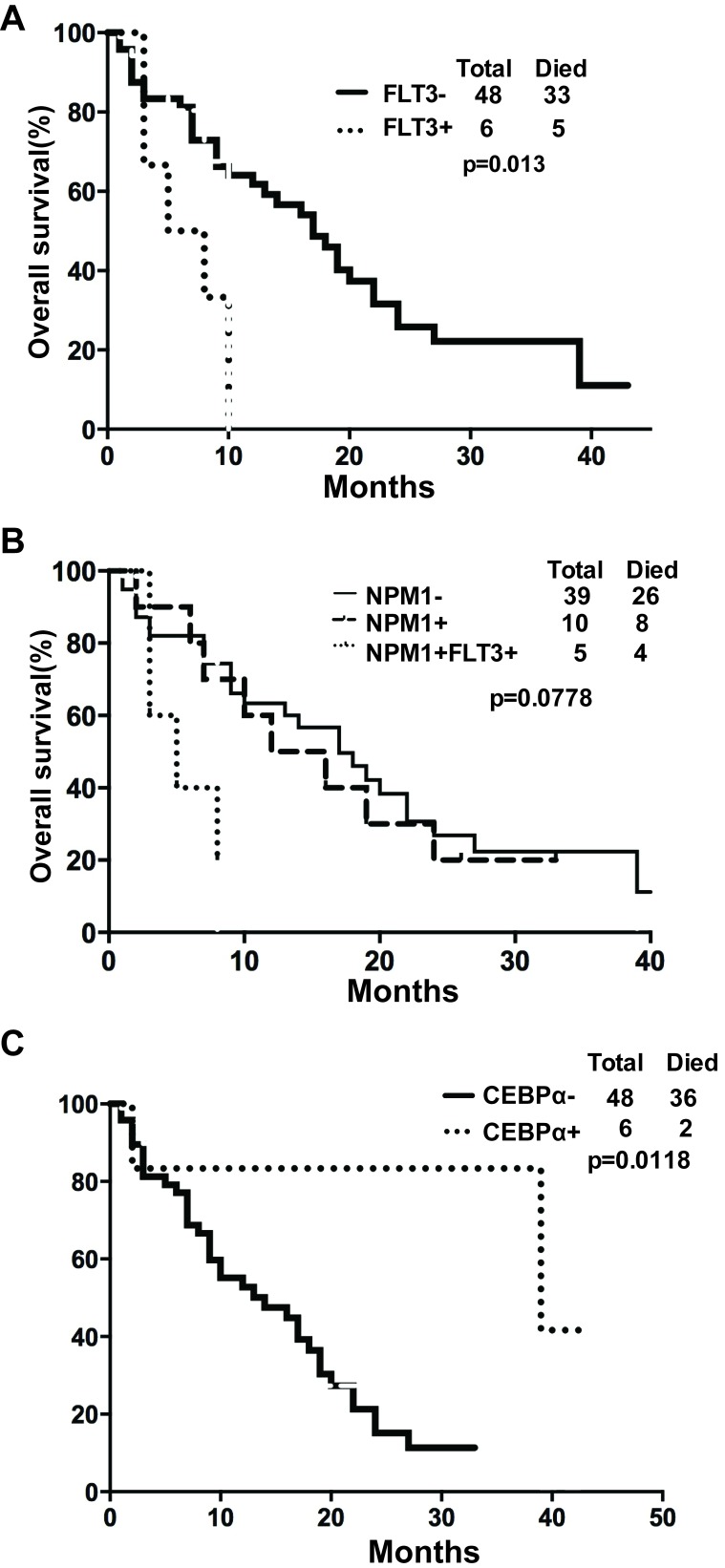
Gene mutation affected the overall survival of elderly patients with AML (A) OS difference in patients with or without *FLT3-ITD* mutation; (B) OS difference in patients with or without *NPM1* mutations; (C) OS difference in patients with or without *CEBPα* mutations.

To investigate whether several known gene mutation might affect the OS of elderly AML patients under D-CAG treatment, we further analyzed the OS of D-CAG treated elderly AML patients by gene mutation, such as: Internal tandem duplications (ITDs) of the FMS-like tyrosine kinase 3 gene (*FLT3*), nucleophosmin (*NPM1*) mutation and CCAAT/enhancer binding protein alpha (*CEBPα*) mutation [15]. *FLT3-ITDs* are located in exons 14 and 15 of the *FLT3* gene and show a broad variation in the position of their insertion sites, as well as in number and sizes of the duplicated fragments [16]. The most common *NPM1* mutations include type A mutations (TCTG) in 80%, followed by type B (CATG) and type D (CCTG) mutations in about 10%, and a spectrum of other mutations accounting for 10% of cases [17]. Two types of *CEBPα* mutations are predominant. N-terminal frame-shift mutations lead to loss of translation of the full-length 42 kD *CEBPα* and the overexpression of a truncated, dominant negative 30 kD *CEBPα*. C-terminal in-frame insertions/deletions prevent homo-dimerization or hetero-dimerization of *CEBPα* [18]. In our study, we found that patients without *FLT3-ITD* mutation have a survival advantage in compared with those with *FLT3-ITD* mutation (*p*=0.013, 95% CI=0.0296 to 0.660) (Fig. 4A). A median OS for patients with wild-type *NPM1* or with mutations in *NPM1* only or with mutations in *NPM1* and *FLT3-ITD* was 17, 15 and 5 months, respectively (*p*=0.0778) (Fig. 4B). A significant OS difference was also observed in patients with *CEBPα* mutations vs. without *CEBPα* mutations (*p*=0.012, 95% CI=1.287 to 7.551) (Fig. 4C).

### Prognostic factors for overall survival in elderly patients with AML

In an exploratory subset analysis using a multivariate Cox proportional Hazards model, adverse cytogenetics (p=0.0234), poor performance status (ECOG PS of 3, p=0.0064), HCT-CI (p=0.0414) but not age, gender, WBC at diagnosis, or percentage of BM blasts (50%), were identified as the independent adverse prognostic factors (Table 2).

**Table 2:** Prognostic factors for survival identified by multivariate analysis in older patients with AML

Characteristics	Groups	HR	95%CI	P value
Gender	Female	0.6923	0.08417 to 1.300	0.5293
	Male	1		
Age (years)	70	1.189	0.7194 to 1.966	0.4994
	60-69	1		
WBC	≥30	1.194	0.6375 to 2.237	0.5794
	<30	1		
BM Blasts	≥50	1.122	0.6700 to 1.878	0.6622
	<50	1		
Karyotype	intermediate	0.3946	0.1766 to 0.8818	0.0234
	poor	1		
ECOG	≥2	2.099	1.232 to 3.578	0.0064
	0-1	1		
HCTCI	0-2	0.5398	0.2985 to 0.9762	0.0414
	3-6	1		

Abbreviations: BM, bone marrow; ECOG, Eastern Cooperative Oncology Group; CI, confidence interval; HCTCI, hematopoietic cell transplantation comorbidity index ; HR, hazard ratio; WBC, white blood count;

### Treatment-related toxicity

### Serious Adverse Events

Overall, D-CAG was well tolerated. Table 3 showed the most frequent adverse events observed during induction therapy. The most common grade 3 and 4 toxicities included thrombocytopenia and neutropenia, while the incidence of non-hematological toxicities was low. Myelosuppression was commonly observed, and febrile neutropenia occurred in 92.3% of patients.

**Table 3:** Most frequent side effects during induction therapy

Side effect	Grade II No. Of patients (%)	Grades III/IV No. Of patients (%)
Neutropenia	13 (14.3%)	78 (85.7%)
Thrombocytopenia	6 (6.6%)	85 (93.4%)
Febrile neutropenia	57 (62.6%)	27 (29.7%)
Nausea, Vomiting	13 (14.3%)	-
Hyperglycemia	20 (22.0%)	5 (5.5%)
Acute pancreatitis	-	1 (1.1%)
Intractable hiccup	-	1 (1.1%)
Skin rashes	3 (3.3%)	-
Liver dysfunction		
Eleated enzymes	13 (14.3%)	2 (2.2%)
Increasing bilirubin level	7 (7.7%)	-
Creatinine elevation	1(1.1)%	-
Cardiac dysfunction	-	1 (1.1%)
Spsychiatric symptoms	6 (6.6%)	-

In patients with CR, the median time was 16 days (range, 5-50 days) for platelet recovery and 23 days (range, 16-46 days) for granulocyte recovery. After a single induction, the platelet count in patients recovered briskly to the normal range, platelets rose and peaked above 500×10^9^/L in 20 patients (all responders), and the count returned to the normal range within 7-10 days. During convalescence, no thrombotic complications were observed.

### The factors caused the death of D-CAG treated elderly patients with AML

64 of 91 patients died on study and reasons for patient death included relapsed disease (50.6%), severe infections (16.5%), refuse to treatment after the first cycle (22%), refractory disease (20.9%), cardiac dysfunction (3.3%), and intracranial hemorrhage (2.2%), multiple reasons (6.6%). Within 4 weeks from the beginning of treatment, 4 patients (4.4%) with ECOG 3 died of severe infection, cardiac dysfunction or intracranial hemorrhage. Of the 70 responding patients, 16 remain CR during follow-up, 51 relapsed in remission (19 relapsed due to discontinued further chemotherapy), 3 died of consolidation chemotherapy.

## DISCUSSION

This phase II study represents, to the best of our knowledge, the first reported prospective trial investigating the use of decitabine in combination with G-CSF, low-dose aclarubicin and cytarabine as the first-line therapy for elderly patients with AML. It was carried out to evaluate the efficacy of D-CAG combination therapy and assess the adverse events in those elderly patients with AML.

Several rationales guide us to design the D-CAG regimen to treat elderly AML patient. DNA methylation was dose dependent with a plateau and correlated with response to decitabine at low (5-20 mg/m^2^/d) but not high doses [19]. DNMT1 depletion by decitabine is S-phase dependent, and is more extensive in actively cycling cells [20]. G-CSF priming induced G0/G1 phase leukemia cells into S phase and caused leukemia cells response to decitabine. In addition, the combination of decitabine and cytarabine showed an additive or synergistic effect on cell death in human leukemia cell lines *in vitro* [7]. Those previous studies suggest that D-CAG regimen may achieve better outcomes in elderly AML patients. Our study showed that decitabine in combination with CAG was well tolerated and effective with CR of 77.7% in elderly patients with previously untreated AML. Our study also revealed a CR of 76.3% and median survival of 10 months, while only 2 patients (4.76%) died within 8 weeks in 42 patients ≥70 years of age. The patients aged ≥70 years benefit more from decitabine in combination with low-dose CAG in comparison with patients from our previous study. With CAG treatment, the ORR and CR were 72.0% and 58.0%, respectively [14]. The median OS was 14 months. In comparison, with D-CAG treatment, ORR and CR were 82.4% and 64.7%, respectively, after 1 cycle of therapy. The ORR in patients aged <70 years was 83.0% and 81.6% in patients aged ≥70 years. There was a significantly longer median OS in patients with response (16 months). Compared to cytarabine-based intensive chemotherapy, our D-CAG-based intensive chemotherapy also showed a higher CR and a longer median survival. A recent study of the cytarabine-based intensive chemotherapy on 446 patients ≥70 years of age with AML obtained a CR of 45%, and a median survival of 4.6 months in all patients, 13.8 months in CR patients, and 8-weeks mortality was 36% [3].

Our study also showed that CR might be an important index to predict the survival probability of elderly patients with AML. In this phase II trial, the survival probability of patients with CR was significantly higher than that of patients without response to D-CAG treatment. These results were comparable to other studies, suggesting that CR might be required for prolonged survival [21].

An interesting finding from this study was that the CR rate in patients with poor karyotype was similar to that in other patients. Consistent with results from an earlier study, there was a clear benefit for patients with both CR and CCyR after induction of chemotherapy [22,23]. Similarly, another study showed that 19/61 (31.2%) MDS patients with clonal chromosomal abnormalities achieved major cytogenetic responses after a median of > 2 cycles (3 cycles) of treatment with low-dose decitabine and had a prolonged OS. Those studies suggested that decitabine induction might be important for patients with poor karyotype. We also found that our patients with “high-risk” chromosomal abnormalities, such as chromosome 7 abnormalities and complex abnormalities, tend to have a longer OS (8.5 months) than that previously reported [14,24]. Decitabine may inhibit those chromosomal abnormalities induced DNA hypermethylation and inactivated genes and cause the longer OS of AML patients.

The prognostic impact of *NPM1*, *CEBPα* and *FLT3-ITD* mutations, was first established by cytogenentic analysis in AML patients who were <60 years of age [30]. Similarly, the relative favorable effect of *NPM1* mutation and *CEBPα* mutation and the unfavorable effect of *FLT3-ITD* mutation were also manifested in elderly AML patients [25-27]. However, there has not been any favorable effect in elderly patients with the *NPM1* mutation, in whom the 2-year OS rate was 19% in a combined SWOG/MRC data analysis [28]. Our study showed that the relative favorable OS was achieved in patients with *CEBPα* mutations, while the outcome in patients with *FLT3-ITD* was significantly poorer. Interesting, *NPM1* mutation itself did not lead to a better survival. These results should be explained with caution, as the patient's sample pool in each group was relative small. Therefore, the role of *NPM1* mutation or other gene mutations, such as *IDH1*, *IDH2* and *TET2* et.al, in the response of elderly AML patients to D-CAG needs further in-depth investigation.

Our results also suggested that clinical benefit from decitabine in combination with low-dose CAG treatment might be linked to the ability to suppress the abnormal clone. However, we found that decitabine alone or in combination with other agents failed to eradicate the abnormal clone. In our study, 11 out of 18 patients with CCyR had a cytogenetic relapse (6 BM relapse and 1 extramedullary relapse). These results were consistent with earlier findings, which suggested that the initial cytogenetic clone can relapse upon D-CAG treatment and D-CAG regimen may not eradicate the abnormal clone [29,30].

In conclusion, decitabine in combination with low-dose CAG treatment appeared to be a feasible, safe and effective for elderly patients with AML. Early results of this prospective trial demonstrated the effectiveness of decitabine-CAG combination and, thus far, limited treatment-related adverse effects in elderly patients. Additional follow-up and more robust phase 2 studies are needed to assess the efficacy and safety of D-CAG-based chemotherapy in elderly patients with AML.

## MATERIALS AND METHODS

### Study population

For this multi-center, phase II study, we recruited elderly patients with AML from 11 medical centers in China from October 2010 through March 2013. This study was registered at www.chictr.org as ChiCTR-ONC-11001700. Patients aged ≥60 years with newly diagnosed *de novo* or secondary AML according to the International Working Group (IWG) criteria [31] who refused or were not candidates for intensive chemotherapy were eligible. All patients had an Eastern Cooperative Oncology Group (ECOG) performance status (PS) of 0-3 with the creatinine level and total bilirubin of ≤2 mg/dL. Exclusion criteria included acute promyelocytic leukemia, another malignancy without remission. Patients must not have previous chemotherapy (other than hydroxyurea) for any myeloid disorder. Comorbidities were assessed using hematopoietic cell transplantation comorbidity index (HCT-CI) [32]. This study was performed in accordance with the Declaration of Helsinki and all patients provided written informed consent. All study procedures and informed consent forms were approved by Institutional Review Board.

### Study design

All patients were treated with decitabine of 15 mg/m^2^ intravenously over 4h for 5 consecutive days (day 1-5) and G-CSF of 300 μg/day (day 0-9) for priming combined with cytarabine of 10 mg/m^2^ q12h for 7 days (day 3-9), aclarubicin of 10 mg/day for 4 days (day 3-6) (D-CAG). The G-CSF priming was discontinued if white blood count (WBC) was >20×10^9^/L. Hydroxyurea was permitted as rescue medication to control WBC to <5.0×10^9^/L and but was discontinued at least 24h before decitabine treatment.

Up to two cycles of induction therapy were allowed if response was not achieved. Patients who achieved CR accepted the next cycle treatment for recovery of hematopoiesis and resolution of all toxicities. Patients who did not achieve CR or partial remission (PR) after the second cycle of induction therapy were offered alternative therapies. Post-remission therapy consisted of 4-6 cycles at the discretion of attending physician, including alternate D-CAG and conventional chemotherapy [cytarabine (100 mg/m^2^ for 7 days) in combination with homoharringtonine (2 mg/m^2^/day for 7 days) or an anthracyclin agent, such as daunorubicin (30 mg/m^2^ for 3 days)]. In patients aged ≥70 years and/or with ECOG≥2, or with hypoplasia/cytopenia, doses were reduced to 70% of scheduled dose levels in subsequent cycles. Treatment was continued until relapse or progressive disease, death, or unacceptable toxicity occurred, or patients/physicians requested the discontinuity. All patients received antimicrobials, supportive care, and transfusions of blood products according to the institutional guidelines.

Treatment responses were defined according to the modified 2003 IWG criteria [31]. Morphologic CR included normalization of bone marrow blasts (≤5% blasts) and peripheral blood counts (absolute neutrophil count ≥1.0×10^9^/L and platelet >100 ×10^9^/L). PR was defined as morphologic CR and 5-15% blasts with a decrease of at least 50% of total bone marrow blasts. Time to hematopoietic recovery was measured from the last day of chemotherapy to the time when the neutrophil count was >0.5×10^9^/L and platelet count was more than 20×10^9^/L. All other patients were considered as non-responders.

Conventional cytogenetic evaluation was also performed after induction therapy. The overall survival (OS) was measured from the time at beginning of the study to death (any cause). Disease-free survival (DFS) was calculated from the date of remission to an event, including resistance, PR, relapse, or death. Early induction mortality within 4 weeks was calculated.

Toxicities were assessed according to Common Terminology Criteria For Adverse Events (CTCAE) version 3.0. Safety was assessed using adverse events, physical examinations, vital signs, and central laboratory assessments.

### Cytogenetic and mutation analysis

Bone marrow (BM) cells were harvested directly or after 1-3 days of unstimulated culture, as described previously. Metaphase cells were banded via an improved heat treatment and R-banding method. Genomic DNA was isolated from BM specimens. Mutation analysis of three relevant molecular marker genes (*NPM1*, *CEBPα* and *FLT3-ITD*) was carried out as described previously [15]. The mutation was determined by collection of the mutated polymerase chain reaction fragment followed by sequencing. The primers used to detect the gene mutation are listed. *NPM1* mutation forward primer: TTAACTCTCTGGTGGTAGAATGAA, *NPM1* mutation reverse primer: CAAGACTATTTGCCATTCCTAAC; *FLT3-ITD* forward primer: GCAATTTAGGTATGAAAGCCAGC, *FLT3-ITD* reverse primer: CTTTCAGCATTTTGACGGCAACC; *CEBPα* mutation forward primer: TCGGCCGACTTCTACGAG, *CEBPα* mutation reverse primer: GCTTGGCTTCATCCTCCTC; *CEBPα* mutation forward primer: GAGGAGGATGAAGCCAAGC, *CEBPα* mutation reverse primer: GTTGCCCATGGCCTTGAC.

### Statistical analysis

Survival curves were plotted by the Kaplan-Meier method and compared by using the log-rank (Mantek-Cox) test stratified by baseline age, cytogenetic risk, and response after treatment. In order to analyze the related impact of each variant on survival, hazard ratios (HRs) and 95% confidence interval (CI) were calculated by using a Cox proportional hazards model stratified by gender, disease, age, cytogenetic risk, WBC at diagnosis, BM blasts, ECOG PS and HCT-CI, respectively. Differences in subgroups by different covariates were evaluated by using the chi-square test for nominal values. A *p* value <0.05 was considered significant.
